# Profiling microRNAs in *Eucalyptus grandis* reveals no mutual relationship between alterations in miR156 and miR172 expression and adventitious root induction during development

**DOI:** 10.1186/1471-2164-15-524

**Published:** 2014-06-25

**Authors:** Aviv Levy, David Szwerdszarf, Mohamad Abu-Abied, Inna Mordehaev, Yossi Yaniv, Joseph Riov, Tzahi Arazi, Einat Sadot

**Affiliations:** The Institute of Plant Sciences, Volcani Center, P.O. Box 6, Bet-Dagan, 5025000 Israel; The Robert H. Smith Institute of Plant Sciences and Genetics in Agriculture, The Robert H. Smith Faculty of Agriculture, Food and Environment, The Hebrew University of Jerusalem, Rehovot, 7610001 Israel; Syngenta Seeds – R&D, Valle de Azapa Km 17, Arica, Chile

**Keywords:** miRNA, Eucalyptus, Juvenile to mature phase change, Adventitious roots

## Abstract

**Background:**

The change from juvenile to mature phase in woody plants is often accompanied by a gradual loss of rooting ability, as well as by reduced microRNA (miR) 156 and increased miR172 expression.

**Results:**

We characterized the population of miRNAs of *Eucalyptus grandis* and compared the gradual reduction in miR156 and increase in miR172 expression during development to the loss of rooting ability. Forty known and eight novel miRNAs were discovered and their predicted targets are listed. The expression pattern of nine miRNAs was determined during adventitious root formation in juvenile and mature cuttings. While the expression levels of miR156 and miR172 were inverse in juvenile and mature tissues, no mutual relationship was found between high miR156 expression and rooting ability, or high miR172 expression and loss of rooting ability. This is shown both in *E. grandis* and in *E. brachyphylla,* in which explants that underwent rejuvenation in tissue culture conditions were also examined.

**Conclusions:**

It is suggested that in these *Eucalyptus* species, there is no correlation between the switch of miR156 with miR172 expression in the stems and the loss of rooting ability.

**Electronic supplementary material:**

The online version of this article (doi:10.1186/1471-2164-15-524) contains supplementary material, which is available to authorized users.

## Background

Plant species gradually mature through four phases: (1) embryonic, (2) postembryonic juvenile vegetative, (3) mature vegetative, and (4) mature reproductive [1, 2]. The most pronounced maturity trait is flowering capacity, but other traits, such the ability to form adventitious roots (ARs), are modified during the phase changes [2–6]. In many woody plants, rooting ability is expressed at the base of the stem, which retains juvenile characteristics throughout the plant life, and is sharply or gradually lost upon transition to the mature phase [2].

Initial indications of the molecular control of the vegetative phase change (phase 2 to 3 above) came from analyses of maize (*Zea mays*) mutants. The *Teopod1* and *Teopod2* mutations led to extended expression of juvenile traits, among which was an increase in the number of nodes bearing ARs [7]. In contrast, the *Glossy15* mutation in maize accelerated the appearance of adult characteristics in juvenile leaves [8, 9]. Later, the *Glossy15* gene, that encodes APETALA2-like [10], was found to be regulated by miR172 [11, 12]. *Corngrass1* (*Cg1*) is a maize mutant in which juvenile traits are retained during the adult reproductive phase [13]. The molecular basis of *Cg1* is overexpression of miR156, concomitant with lower levels of miR172 [13]. Similarly, tomato (*Solanum lycopersicum* cv. ‘Ailsa Craig’) plants overexpressing miR156 exhibited increased development of stem ARs [14]. MiR156 and miR172 have been found to coordinate the juvenile-to-mature phase change in *Arabidopsis*. miR156 targets the transcription factors *SQUAMOSA PROMOTER BINDING PROTEIN LIKE (SPL) 9* and *10* that promote miR172b transcription, which in turn exhibits a regulatory feedback loop with its targets *TOE1* and *2* [12].

miR156 and miR172 have been shown to have similar expression patterns in various woody plants, including *Acacia confusa*, *Acacia colei*, *Eucalyptus globulus*, *Hedera helix*, *Quercus acutissima,* and *Populus* x *canadensis*, during the juvenile-to-mature phase change [15]. Overexpression of miR156 in transgenic poplar (*Populus* x *canadensis)* reduced the expression of miR156-targeted *SPL* genes and miR172 and prolonged the juvenile phase [15]. Taken together, these data indicate that miR156 is an evolutionarily conserved regulator of the vegetative phase change in both herbaceous and woody plants, whereas miR172 is a regulator of maturity [12, 15].

Other miRNAs have been shown to be directly involved in AR formation in Arabidopsis, with *ARF17,* a target of miR160, being a negative regulator, and *ARF6* and *ARF8*, targets of miR167, being positive regulators [16]. These transcription factors are also involved in regulation of the cross-talk between auxin and jasmonic acid pathways during AR formation [17].

The juvenile-to-mature phase change of *Eucalyptus grandis* was previously characterized [18]. It was shown that rooting capability was higher in cuttings excised below node 5, termed juvenile, than in those excised above node 15, termed mature [18]. The sequencing of *Eucalyptus grandis* genome has made the plant a valuable experimental system. In the present study, a comprehensive profiling of miRNAs was carried out and the expression of miR156 and miR172 was studied in relation to the loss of rooting ability in juvenile and mature cuttings of *E. grandis* and *E. brachyphylla.*

## Results

### Profiling of miRNA expression

To obtain a maximum spectrum of miRNAs expressed in *E. grandis*, total RNA was extracted from 14-day-old intact seedlings and subjected to miRNA discovery-oriented deep sequencing. After filtering, a large number of the reads matched t/rRNA sequences (Table 1). Analysis by the ShortStack application [19] revealed 40 known and 8 novel microRNAs in the *E. grandis* RNA sample (Additional file 1: Table S1). Among the conserved microRNAs were miR156, miR157, miR160, miR164, miR166, miR167, miR171, miR172, miR319, miR390, miR394, miR395, miR396, miR408, and miR828. BLAST results for the predicted targets of conserved miRNAs identified in *E. grandis* (Additional file 2: Table S2) indicated that most of them were similar to predicted targets documented in other plants. The predicted targets for the novel miRNAs identified by the ShortStack analysis are listed in Additional file 3: Table S3.

**Table 1:** Sequence data analysis

	Total	Non redundant
**Input**	6,989,302	72,258
**Valid sequence filter**	6,891,830	67,710
**t/rRNA filter**	2,111,579	49,097
**Genome filter**	1,273,259	32,300

A series of digital filters were employed on the raw readings to discard sequences irrelevant to microRNA identification.

Northern blot analysis was performed to determine possible changes in miRNA expression during induction of AR formation in juvenile and mature cuttings (Additional file 4: Figure S1). Cuttings were excised from different stem sections of stock plants of the same chronological age. Juvenile cuttings were excised from 10 to 15 cm above the ground, and mature cuttings from about 2 m above the ground. Under these experimental conditions, AR primordia start to form, after 3 to 9 days only in juvenile cuttings, confirming earlier studies showing that shoots in close proximity to the root system retain juvenile traits [18]. No consistent differences were found between juvenile and mature cuttings in the expression of miR160, 164, 166, 167, 171, 396 and 397, during the first 9 days after excision and auxin application. In addition, the expression levels of the corresponding predicted targets of these miRNAs were analyzed in the above cuttings by the nanostring method (Additional file 5: Table S4). No consistent differences were found between them, except for NAC1 which was gradually upregulated only in juvenile cuttings during AR induction (Additional file 5: Table S4).

### Expression of miR156 and miR172 in relation to the loss of rooting ability

**Figure 1:**
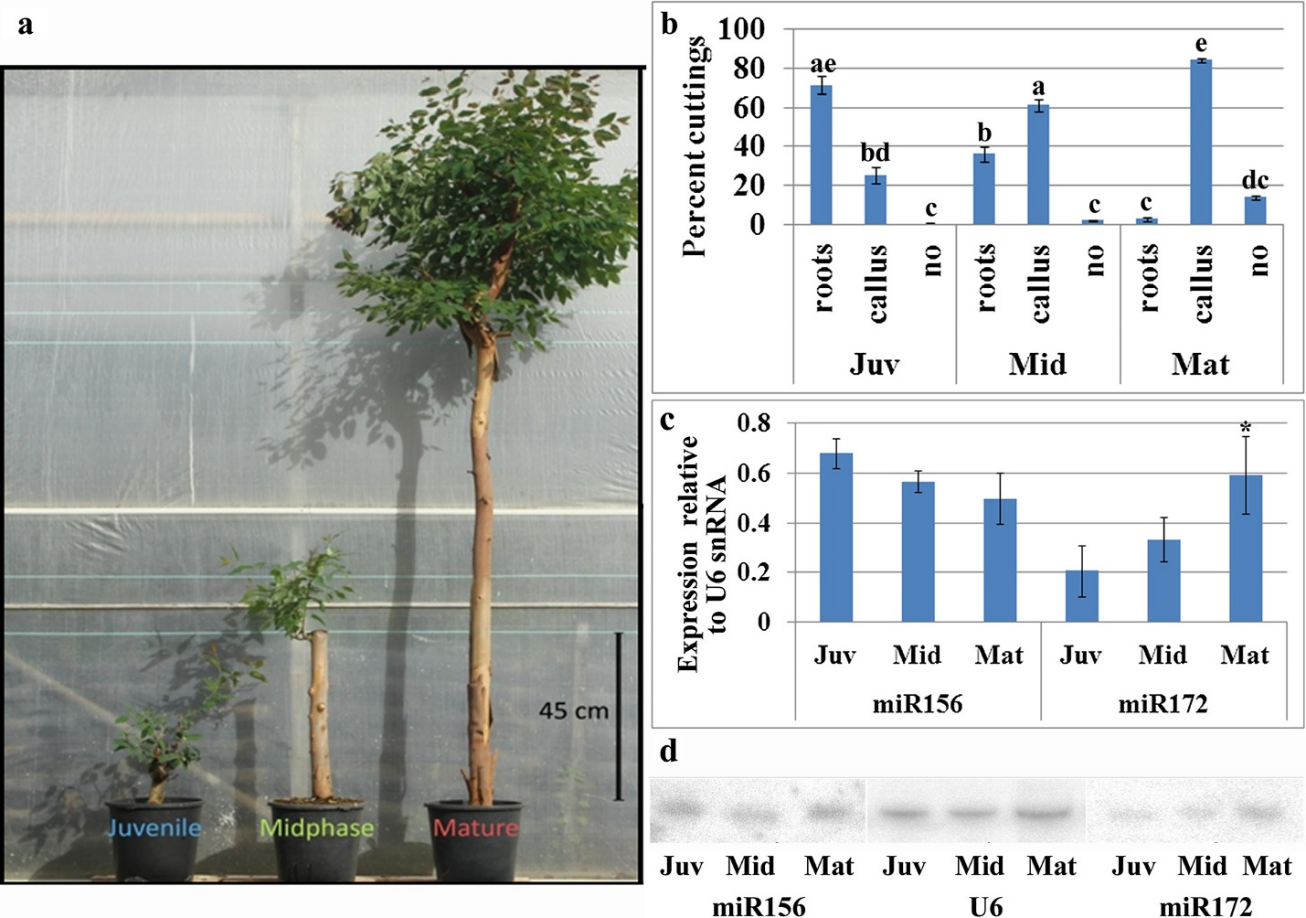
**Comparison of rooting ability and miR156 and miR172 expression in juvenile (Juv), midphase (Mid) and mature (Mat) cuttings. Cuttings were excised 15, 45 or 200 cm above the ground (a), treated with auxin (20 sec deep in 6gr/l K-IBA) and incubated on rooting tables. (b)** Percent rooting and callus formation after 30 days. Bars show averages and standard error. Bars with different letters are statistically different as determined by Scheffe analysis p < 0.05. **(c,d)** RNA was prepared from the bottom of the cuttings and miR156 and miR172 expression analysis was performed by Northern blots: **(c)** average and standard error of three experiments, asterisk show statistically significant difference as determined by Scheffe analysis p < 0.05. **(d)** representative gels.

Changes in miR156 and miR172 expression have been found to be key regulators of the juvenile-to-mature phase change [12]. Here, the pattern of miR156 and miR172 expression was determined in the tissue giving rise to AR formation in two plant systems. The first was *E. grandis*, in which a third type of cutting was excised 45 cm above the ground, termed midphase (Figure 1a). The rooting potential was found to decrease with increasing distance from the ground from which the cuttings were collected (Figure 1b). In parallel to the loss of rooting potential, an increase in callus formation was observed, in agreement with previous findings in other woody plants [20–22]. While miR156 expression declined slightly during maturation, miR172 exhibited an opposite pattern, with significant elevated levels in mature cuttings compared to juvenile ones (Figure 1c,d), in agreement with previous findings in other trees [15]. Interestingly, in midephase, the decline in expression of miR156 and the increase in that of miR172 compared to juvenile cuttings was not significant, but the difference in rooting ability and increase in callus formation between these cutting was statistically significant (Figure 1b). We concluded that the loss of rooting ability in *E. grandis* may precede a significant decline and increase in miR156 and miR172 expression, respectively, in the tissue giving rise to AR formation.

### Expression of miR156 and miR172 in relation to loss of rooting ability in *E. brachyphylla*

**Figure 2:**
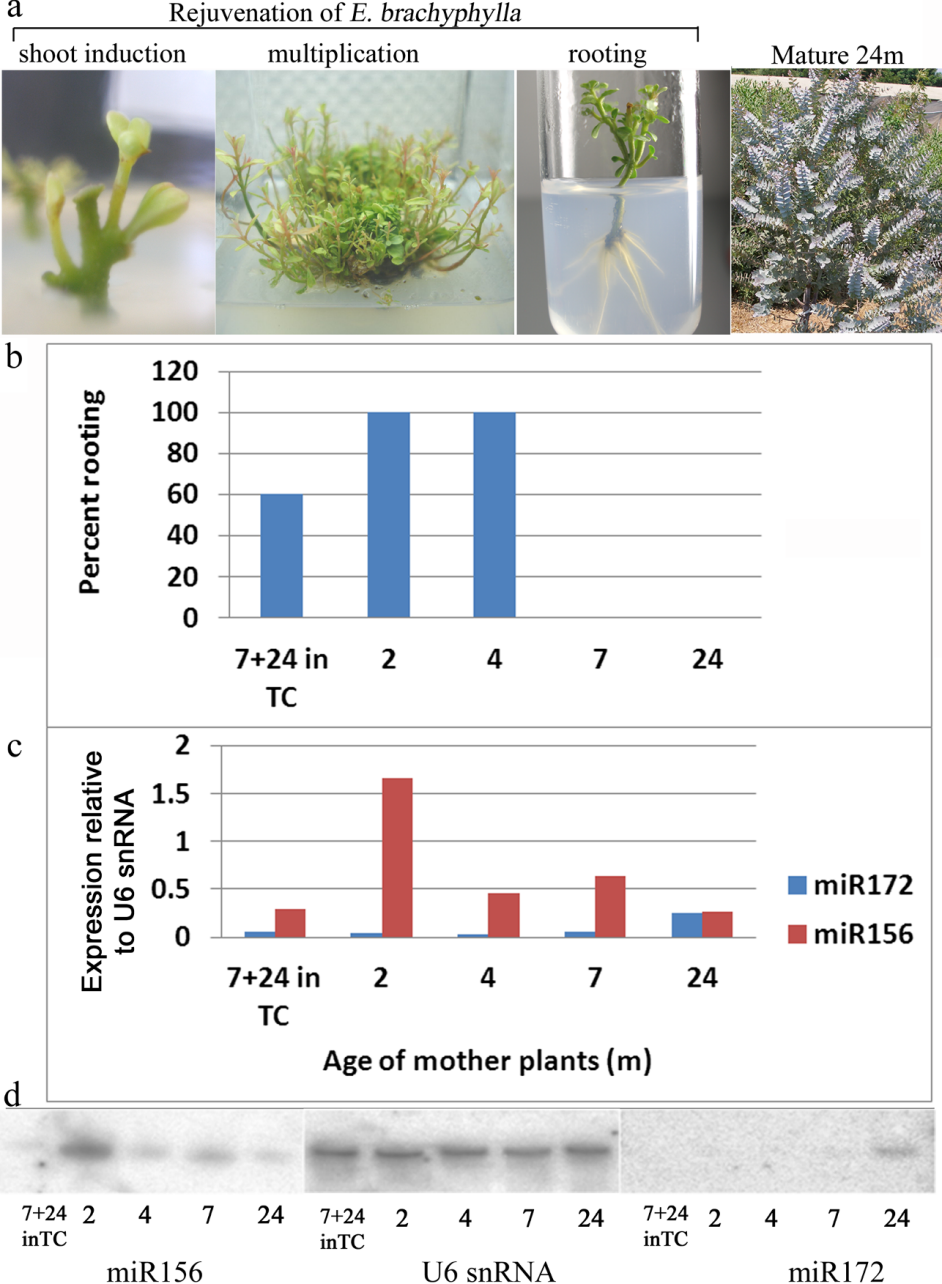
**Rejuvenation of** ***E. brachyphylla*** **and determination of miR156 and miR172 expression. (a)** Sections from a 7-month-old tree, which had lost its rooting ability, rejuvenated in tissue culture (TC). **(b)** Rooting percentage in *E. brachyphylla* plants at different ages. **(c,d)** Analysis of miR156 and miR172 expression: **(c)** quantitative analysis of the gels in **(d)**. Rooting was determined using 50 cuttings and RNA was prepared from 30 cuttings from each age.

We examined the relationship between miR156 and miR172 expression and the loss of rooting ability in another *Eucalyptus* species, *E. brachyphylla*. This species is an ornamental plant characterized by bluish leaves. *Eucalyptus* trees with bluish leaves exhibit a loss of rooting ability early in their juvenile phase [23]. Comparison of the rooting ability of *E. brachyphylla* plants at different ages revealed that rooting ability could be detected up to 4 month age, lost at 7 month age, but could be restored by rejuvenation in tissue culture (Figure 2). Concomitantly, miR156 was highly expressed at the base of the above cuttings at 2 month age, after which it declined, whereas miR172 expression was barely detectable in plants younger than 24 months (Figure 2). To restore the rooting ability of mature *E. brachyphylla*, rejuvenation under tissue-culture conditions was performed (Figure 2). Interestingly, the expression of both miRNAs was very similar in both 7 month age plants and in plantlets that originated from similar plants, and kept in rejuvenation conditions for 24 months for restoration of rooting ability. The data indicate that, rooting ability was not mutually related to a high miR156 expression, and loss of rooting ability was not mutually related to high miR172 expression in the tissue giving rise to ARs.

### Profiling the expression of miR156 and miR172 and their predicted targets during induction of AR in *E. grandis*

**Figure 3:**
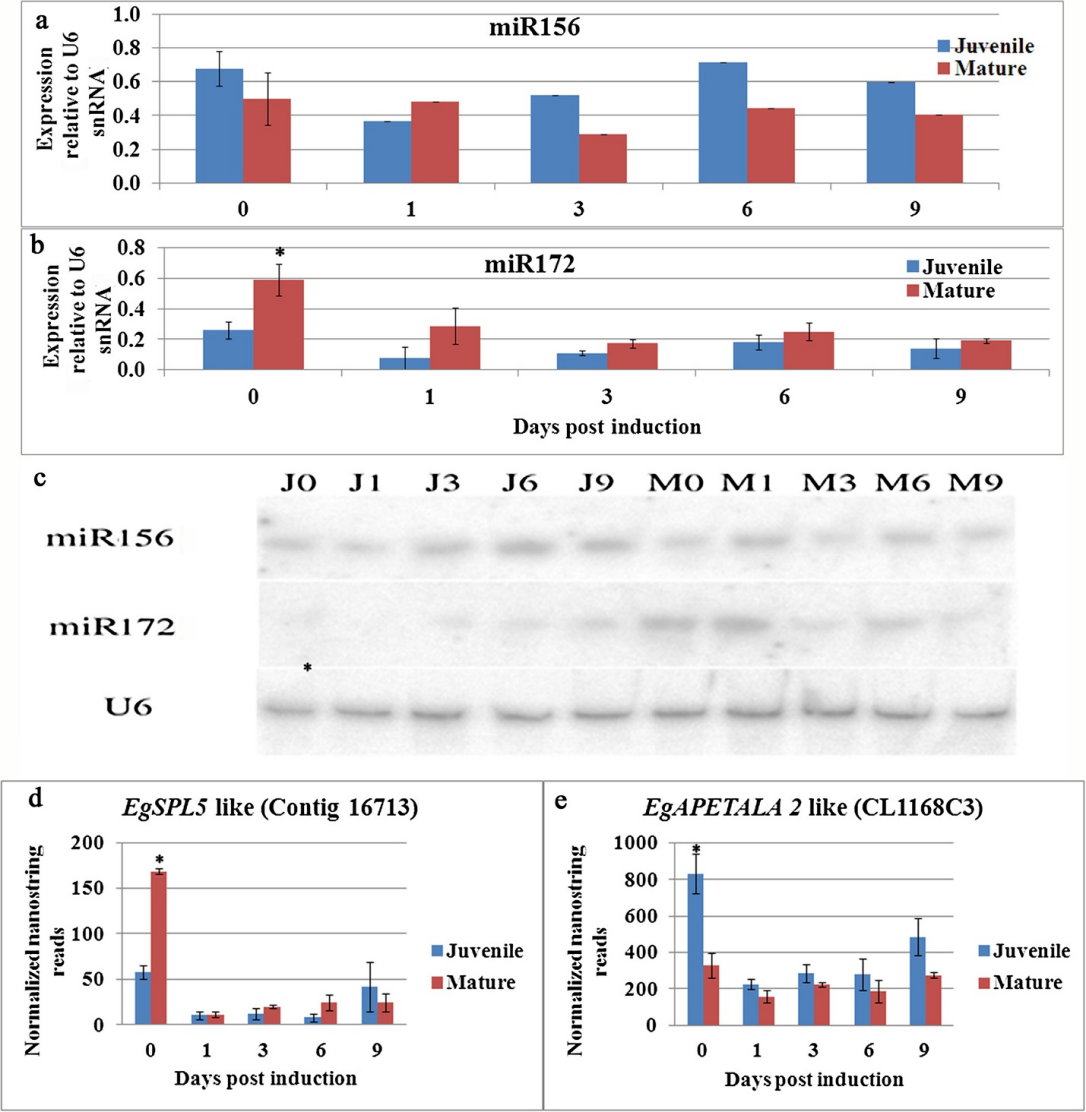
**Analysis of miR156, miR172 and their predicted targets in juvenile and mature cuttings during induction of adventitious root formation.** Cuttings were excised, treated with auxin and incubated on rooting tables for the indicated times. RNA samples were prepared and the expression of microRNAs and predicted targets was determined by northern blot and nanostring analysis, respectively from three independent biological repeats. Quantitaive analysis of **(a)** miR156 expression, and **(b)** miR172 expression. **(c)** Representative gels: J, juvenile; M, mature. Quantitative analysis of the predicted targets of **(d)** miR156 and **(e)** miR172. Asterisks show statistically significant difference as determined by Scheffe analysis p < 0.05.

To determine the functionality of miR156 and miR172 during the induction of AR formation, their expression was monitored at 0, 1, 3, 6 and 9 days after rooting was induced. In addition, the expression of a representative predicted target of each of these miRNAs was determined in the same RNA samples (As in Additional file 5: Table S4). In agreement with previous studies [24, 25], Figure 3 shows that while the expression of miR156 was higher in juvenile cuttings than in mature ones on day 0, expression of its predicted target *EgSPL5* was significantly lower in the juvenile cuttings. Similarly, while the expression of miR172 was lower in juvenile cuttings than in mature ones on day 0, the expression of its predicted target *EgAPETALA 2* was higher in the former, in agreement with previous data [9–11]. Interestingly, miR156 expression was not affected during the first 9 days of induction, whereas that of *EgSPL5* in mature cuttings decreased after 1 day and remained low. Similarly, the expression of miR172 in mature cuttings and that of *EgAPETALA2* in juvenile cuttings decreased after 1 day and remained low until day 9. These changes led to equalization of the expression of both miRNAs and predicted targets in juvenile and mature cuttings during 3 to 9 days after rooting induction, although only juvenile cuttings formed roots under these conditions.

## Discussion

The change from juvenile to mature phase in plants is a gradual process; culminating in the most recognizable developmental process—flower production. However, prior to the change to the reproductive phase, there is a change from juvenile to adult vegetative phase [2]. The characteristics of this transition are more subtle and differ from one plant species to the other. For example, in *Arabidopsis*, juvenile characteristics include round and small leaves, lacking trichomes on their abaxial side [26]. The juvenile characteristics in maize include leaf epidermal wax and nodes bearing ARs [7]. It has been shown that miR156 is a regulator of this vegetative phase change in both *Arabidopsis* and maize. Overexpression of miR156 in *Arabidopsis* led to increased number of small and round leaves without abaxial trichomes [27]. In maize, it led to increased number of leaves with epidermal wax and nodes with ARs [7, 13]. Furthermore, miR156 expression has been found to be correlated with changes in leaf shape in different woody plants [15]. In agreement with previous studies, our observations show that in both *E. grandis* and *E. brachyphylla*, a relatively high level of miR156 is expressed in juvenile tissues, although no marked changes in leaf shape between the juvenile and mature phases were observed in these *Eucalyptus* trees, as reported for example for *E. globulus* [15]. Of note, while the expression of miRNAs was previously determined in the leaves or shoot apices [12, 15], we analyzed their expression in the stems. This might explain why we could not relate the timing of the loss of rooting ability to the reduction in miR156 expression. In *E. grandis*, a significant decline in rooting ability was observed as the distance of the cutting source from the ground increased, although only a subtle decline in miR156 expression was observed in the same plant samples. In *E. brachyphylla*, a high expression of miR156 was observed in 2-month-old plants. This expression significantly decreased in 4-month-plants and thereafter remained more or less constant in 7- and 24-month-old plants, as well as in 7-month-old plants that were rejuvenated in tissue culture for 24 months. However, rooting ability was 100% in 2- and 4-month-old plants, 0% in 7- and 24-month-old plants and 60% in the rejuvenated plants. The uncoupling of miR156 expression and AR forming ability in these *Eucalyptus* trees is in agreement with findings obtained from *Acacia* species. While in *Acacia colei* the leaf change was correlated to the reduction in miR156 and induction of miR172 [15], in *A mangium* the leaf change was not correlated to significant differences in rooting abilities [28–31]. Therefore, regulation of AR formation is still unclear. On one hand, cuttings excised close to the root system possess a high rooting potential [2], suggesting the transport of rooting signals from the roots to the stems. On the other hand, overexpression of miR156 in maize *Corngrass1* mutant led to more nodes with ARs [13], and denser aerial roots along the tomato stems [14]. Expression of miR156 also extended the tuber-forming potential to distal axillary buds in potato and tomato [32]. But regulation of miR156 expression in *Arabidopsis* has been shown to be root-independent and leaf-dependent [33], while, rooting inhibitors have been found in leaves of mature *Eucalyptus* trees [18]. It might therefore be suggested that the regulation of rooting and the regulation of miR156 are uncoupled, but nevertheless downstream effects of miR156 are relevant for AR formation. The precise relationship between miR156 expression and AR formation in *Eucalyptus* has yet to be determined.

The expression of miR172 has been shown to be regulated by miR156 via SPL9 and SPL10 [12]. Therefore, the decline in miR156 expression is expected to occur simultaneously with the increase in that of miR172. Indeed, such a correlation has been shown in *Arabidopsis* [12]. However, a closer look at woody plants revealed a somewhat more complicated picture. When the expression of both miRNAs was analyzed in 1-month-old, and 1-, 4-, and 10-year-old poplar trees, a gradual decline in miR156 expression was found between 1-month and 4-year-old trees, whereas a rise in miR172 expression was only observed in 10-year-old trees, in which there was no further decline in miR156 expression [15]. Similar observations were recorded when samples were taken from shoots at different heights (0.5, 2, and 4 m) or from leaves on the same shoot located at different distances from the main stem. While a gradual decrease in miR156 expression was detected between shoots taken from 0.5 and 2 m above the ground, no detectable change in miR172 expression was observed in these shoots. An increase in miR172 expression was detected only at 4 m above the ground. Similarly, when expression was determined in sequential leaves on the same shoot, a gradual decrease in miR156 expression was observed with increasing distance from the main stem. However, miR172 expression increased only in the last, most distantly located leaf [15]. Therefore, the decline in miR156 expression preceded the rise in miR172 expression. Our results, albeit obtained from a different tissue, the stems, also show no tight correlation between the timing of miR156 downregulation and miR172 upregulation. Taken together, these data suggest that the expression of miRNA156 and miR172 in woody plants is governed by a complex regulation system, which specifically in cuttings, might be also influenced by wounding, the decrease in leaf area, the loss of roots, and a long rooting period under varying environmental conditions.

## Conclusions

It is concluded that in *E. grandis* and *E. brachyphylla*, there is no mutual relationship between easy-to-root cuttings and high miR156 or difficult to root cuttings and high miR172 expression at the base of these cuttings.

## Methods

### Plants

*E. grandis* plants were grown from seeds in a nethouse in 15 liter pots containing peat and tuff (70:30, v/v), drip irrigated and fertilized with 3 liters of Shefer737 liquid fertilizer (ICL Fertilizers, Be’er Sheva, Israel) N.P.K 737 + microelements per m^3^ of water. *E. grandis* plants of the same age were hedged at different heights to promote shoot initiation from specific stem sections for cutting production. Cuttings were treated with 6 g l^−1^ K-IBA (Indole-3-butiric acid, potassium salt) for 20 s and rooted in vermiculite/perlite 1:1, heated to 24°C under constant 90% humidity in rooting tables. Rooting was monitored after 30 days. *E. brachyphylla* cuttings were collected from young seedlings originated from seeds. Seeds were germinated in vermiculite in a temperature-controlled chamber at 23°C and transferred to pots at 1 month age. For rooting tests at 2 month age, excised seedlings served as cuttings, whereas at 4 and 7 month age, cuttings were excised from shoots.

To grow *E. grandis* under sterile conditions, seeds were sterilized in 1% sodium hypochlorite for 5 min and washed three times with sterile ddH_2_O before germinating in 10-cm diameter Petri dishes with 0.8% agar (Sigma) MS (Murashige & Skoog, Duchefa Biochemie, Haarlem, the Netherlands) growth medium at 24°C under a 16-h light/8-h dark cycle. After germination, seedlings were transferred to sterile 50-ml tissue-culture tubes. About 45 days after germination, when the seedlings reached 3 to 4 cm in height, ~3-cm long cuttings were excised and transferred to a fresh medium. The medium consisted of 0.8% agar, full-strength Woody Plant Growth medium (Duchefa Biochemie, Haarlem, the Netherlands) containing 3% (w/v) sucrose, 0.004% (w/v) polyvinylpyrrolidone (PVP; Sigma Aldrich), 50 ppm cysteine (Sigma), and 50 ppm ascorbic acid (Sigma); the pH was adjusted to 5.7 with KOH. Rooting growth medium for cuttings was supplemented with 10 μM sodium nitroprusside (Sigma) [34], 5 μM K-IBA , and 8 μM auxin conjugate [35].

Rejuvenation of *E. brachyphylla:* To avoid endogenous contamination, mother plants were treated with fungicide (2 g l^−1^Daconil) 1 and 2 weeks before the material was introduced into tissue culture. Actively growing branches were immersed in water containing 0.01% (v/v) Triton X-100 immediately after cutting, transferred to the lab, and soaked for 1h in running tap water. Shoots were cut to about 15 cm in length, the leaves were removed, and at least half of each pedicle was left attached to the node. Shoots were surface-sterilized with 70% (v/v) ethanol for 30 s and submerged in a solution containing 1.5% (v/v) NaClO and 0.01% Triton X-100 with constant agitation for 10 min, followed by four full-volume sterile H_2_O washes. Single nodes were cut from the sterile shoots and induced to form multiple shootlets with the following medium: full-strength McCown Woody Plant Media (Duchefa), 8 g l^−1^ agar (Sigma), 30 g l^−1^ sucrose, 400 mg l^−1^ PVP (tissue-culture tested, Sigma), adjusted to pH 5.8 ± 0.1 with 1 N NaOH before autoclaving. Filter-sterilized ascorbate (50 mg l^−1^) and cysteine (50 mg l^−1^) were added along with hormones for induction (for hormone types and concentrations see Additional file 6: Table S5). The medium was replaced every 2 to 3 weeks. Growth conditions were a 16-h photoperiod and, 22 to 25°C. All hormone stocks were prepared at a concentration of 1 mg ml^−1^ (1,000X) and kept at −20°C (up to 6 months). The rejuvenated cultures were maintained by monthly medium replacement for 2 years for gene expression profiling and rooting tests.

### RNA preparation

For deep sequencing, seeds were germinated and plant material samples including roots, stems and leaves were collected 14 days after germination. When cuttings were used, only the 1 cm lower stem section was taken for RNA extraction. All samples were frozen in liquid nitrogen immediately following collection, ground to a fine powder, and stored at −80°C until use. Total RNA was extracted using Plant/Fungi total RNA purification kit (Norgen Biotek, Thorold, Ontario, Canada) according to the manufacturer’s protocol, except that each column was loaded with lysate from 300 mg of plant material instead of ≤50 mg to increase RNA yield. Extracted RNA was checked for concentration and purity in an ND1000 spectrophotometer (Thermo Scientific, Wilmington, DE, USA). Samples with a 260/280 nm absorbance ratio of <1.9 or 260/230 nm absorbance ratio of <1.7 were discarded or further purified with RNeasy Mini Kit (Qiagen, http://www.qiagen.com) according to manufacturer's protocol. RNA samples were kept in the elution buffer at −80°C until use.

### Deep sequencing and analysis

miRNA discovery-oriented deep sequencing was performed by LC Sciences (http://www.lcsciences.com, Houston, TX, USA). Raw sequencing data were processed with the ACGT-miR-v3.5 package (LC Sciences) to filter out adaptors, technical impurities, repetitive sequences, and low-quantity reads (<3) while applying length-cutoff values (15–26 nt), resulting in a nonredundant sequence list. The sequence data was deposited in GEO NCBI (GSE58367, http://www.ncbi.nlm.nih.gov/geo/query/acc.cgi?acc=GSE58367). The ShortStack small RNA gene annotation tool [19], which analyzes small RNA sequencing data by alignment to a reference genome, was used to infer and annotate small RNA genes.

Target prediction was performed by the psRNATarget tool (http://plantgrn.noble.org/psRNATarget) [36]. To improve the available EST (expressed sequence tags) database of *E. grandis*, three publicly available *E. grandis* ESTs were downloaded from The University of Pretoria’s Eucalyptus database server (http://eucalyptusdb.bi.up.ac.za/). These included the Kirst.fa with 190,106; leaf_est.fa with 545,604 and e_grandis_xylem_ests_JGI.fa with 218,133 sequences with average length of 163 (nt) and median length of 108 (nt). The sequences were preclustered with TGICL pipeline version 2.1 (http://sourceforge.net/projects/tgicl/) to form similarity groups, from which the CAP3 sequence assembly program [37] was used to assemble 48,397 contigs and 94,070 singletons with average length of 772 (nt) and median length of 706 (nt). BLAST2GO [38] was then used to annotate sequences longer than 300 bases. This was done by Dr. Shifra Ben Dor from the Bioinformatics Unit, Biological Services, Weizmann Institute of Science, Rehovot, Israel. The new database was implemented into the psRNATarget tool.

### RNA northern blot analysis

RNA blot analysis was performed as previously described [39] with several modifications. A polyacrylamide (1:19 bis: acryamide) gel (15%) (Bio-Rad, https://www.bio-rad.com) was cast in 7 M urea (Sigma) and buffered with 20 mM 4-morpholinepropanesulfonic acid (MOPS; Sigma) using a MiniVE vertical gel system (Huefer, MA, USA) assembled with 9 x 9 cm plates and 1.5-mm spacers, and allowed to solidify for 2 h. The gel was then prerun at 100 V for 15 min in cold 20 mM MOPS-NaOH (pH 7). Deionized formamide (Bio-Rad) was added 1:1 (v/v) to 11 μg of total RNA sample, and 6X loading dye (Fermentas, http://www.fermentas.de/) was then added to samples before they were heated to 95°C for 2 min followed by snap cooling on ice. The gel wells were flushed with running buffer using a syringe just prior to sample loading. The gel was run at 100 V for 2.5 h.

To verify RNA quality on the gel, it was placed in 0.5 μg ml^−1^ fresh ethidium bromide (Hy Laboratories Ltd., Rehovot, Israel), in 20 mM MOPS–NaOH (pH 7) for 10 min and then transferred onto a UV light box: strongly stained bands of >60 nt indicated RNA of acceptable quality. RNA samples were transferred to a Hybond NX nylon membrane (Amersham, http://www.gelifesciences.com) using a Trans-Blot semi-dry electrophoresis system (Bio-Rad) set at 20 V for 40 min. For cross-linking,the nylon membranes were treated with 0.16 M 1-ethyl-3-(3-dimethylaminopropyl) carbodiimide (EDC; Sigma) intercalating agent in 0.13 M 1-methylimidazole-HCl (pH 8) at 60°C for 60 min. The membranes were then thoroughly rinsed with DEPC (diethylpyrocarbonate)-treated water and stored at −20°C until use. Membranes were prehybridized for 1 h with 15 ml of EZ hybridization solution (Biological Industries, Beit-Haemek, Israel) at 42°C. Membranes were then hybridized with 20 pmol oligonucleotides labeled with 20 μCi of γ^32^P ATP (Perkin-Elmer, http://www.perkinelmer.com/) at 42°C overnight in EZ hybridization solution. After hybridization, the membrane was washed three times at 55°C with 2X saline sodium citrate (Sigma) and 0.2% (w/v) sodium dodecyl sulfate (Sigma) solution for 15 min and allowed to dry. Autoradiography was performed with FLA-5000 Phosphorimager (Fuji, Tokyo, Japan). Signal intensity of each band was quantified using ImageJ (http://rsb.info.nih.gov/ij/). U6 small nuclear RNA was used to assess the total amount of RNA loaded in each gel well. Each miRNA probe’s signal was divided by its corresponding U6 signal.

### Expression of predicted target

Expression analysis of selected predicted targets was performed by Nanostring (http://www.Nanostring.com, Seattle, WA, USA). The probes used are listed in Additional file 7: Table S6.

## Electronic supplementary material

Additional file 1: Table S1: *E. grandis* microRNAs analyzed by ShortStack (numbers separated by commas correspond to each sequence when there is more than one). (XLSX 23 KB)

Additional file 2: Table S2: Predicted targets of known *E. grandis* microRNAs. (DOCX 91 KB)

Additional file 3: Table S3: Predicted targets of *E. grandis* novel microRNAs discovered in this study. (DOCX 18 KB)

Additional file 4: Figure S1: Levels of expression of microRNAs during adventitious root formation. (TIFF 7 MB)

Additional file 5: Table S4: Levels of expression of predicted targets during adventitious root formation. (DOCX 21 KB)

Additional file 6: Table S5: Concentrations of hormones used for tissue culture of *E. brachyphylla.* (XLSX 11 KB)

Additional file 7: Table S6: The probes used for the Nanostring analysis. (XLSX 19 KB)

## Abbreviations

AR: Adventitious roots
miR: microRNA
IBA: Indole-3- butyric acid.
