# Supplementary material for: Profiling microRNAs in Eucalyptus grandis reveals no mutual relationship between alterations in miR156 and miR172 expression and adventitious root induction during development
Source: BMC Genomics. 2014 Jun 25;15(1):524. doi: 10.1186/1471-2164-15-524 (PMC4094776; doi:10.1186/1471-2164-15-524)
Supplement: Supplementary file 2 — Additional file 2: Table S2: Predicted targets of known E. grandis microRNAs. (DOCX 91 KB) [file 12864_2014_6229_MOESM2_ESM.docx]

| Target prediction | | | | | Target BLAST | | | | |
| --- | --- | --- | --- | --- | --- | --- | --- | --- | --- |
| miRNA family accession | Target accession | E value | Target start | Target end | Target description | Score | E value | Swiss-Prot accession | Reference of studies describing similar targets |
| miR156;_miR157 | CL7790Contig1 | 1 | 865 | 885 | SPL9_ARATH | 177 | 5.00E-50 | Q700W2.2 | ([Rhoades et al., 2002](#_ENREF_75); [Schwab et al., 2005](#_ENREF_82); [Wang et al., 2011](#_ENREF_90)) |
| miR156;_miR157 | Contig16713 | 1 | 722 | 741 | SPL5_ARATH | 143 | 3.00E-41 | Q9S758.1 |  |
| miR156;_miR157 | Contig7759 | 1 | 1440 | 1460 | SPL12_ORYSI | 224 | 2.00E-64 | A2YGR5.1 |  |
| miR156;_miR157 | CL2094Contig2 | 2 | 407 | 427 | - | | | | - |
| miR156;_miR157 | Contig15230 | 2 | 1128 | 1148 | SPL16_ORYSJ | 192 | 5.00E-54 | Q6YZE8.1 | ([Rhoades et al., 2002](#_ENREF_75); [Schwab et al., 2005](#_ENREF_82); [Wang et al., 2011](#_ENREF_90)) |
| miR156;_miR157 | Contig17662 | 2 | 621 | 640 | SPL4_ARATH | 137 | 5.00E-39 | Q9S7A9.1 |  |
| miR156;_miR157 | Contig3897 | 2 | 1109 | 1129 | SP13A_ARATH | 203 | 3.00E-59 | B9DI20.1 |  |
| miR156;_miR157 | Contig9043 | 2 | 1362 | 1382 | DPNP1_ARATH | 494 | 2.00E-171 | Q42546.1 | - |
| miR156;_miR157 | F7JO6JY01D2W7F | 2 | 240 | 259 | - | | | | - |
| miR156;_miR157 | F7JO6JY01EKKCZ | 2 | 59 | 79 | - | | | | - |
| miR156;_miR157 | CL2094Contig1 | 2 | 289 | 309 | SPL9_ARATH | 177 | 5.00E-50 | Q700W2.2 | ([Rhoades et al., 2002](#_ENREF_75); [Schwab et al., 2005](#_ENREF_82); [Wang et al., 2011](#_ENREF_90)) |
| miR156 | isotig27111 | 2 | 1362 | 1381 | MYB6_ARATH | 278 | 2.00E-91 | Q38851.1 | - |
| miR156 | CL2094Contig2 | 2 | 289 | 308 | - | | | | ([Rhoades et al., 2002](#_ENREF_75); [Schwab et al., 2005](#_ENREF_82); [Wang et al., 2011](#_ENREF_90)) |
| miR160* | CL13284Contig1 | 2 | 1058 | 1077 | - | | | |  |
| miR160 | CL2280Contig2 | 2 | 1058 | 1077 | ARFJ_ARATH | 217 | 7.00E-111 | Q9SKN5.1 | ([Rhoades et al., 2002](#_ENREF_75); [Mallory et al., 2005](#_ENREF_57); [Sorin et al., 2005](#_ENREF_87); [Wang et al., 2005](#_ENREF_91); [Lu et al., 2007](#_ENREF_54); [Gutierrez et al., 2009](#_ENREF_29); [Zeng et al., 2010](#_ENREF_102)) |
| miR160 | Contig2391 | 0 | 722 | 741 | ARFR_ORYSJ | 460 | 0 | Q653H7.1 |  |
| miR160 | CL2833Contig2 | 1 | 489 | 509 | ARFQ_ARATH | 188 | 5.00E-53 | Q84WU6.1 |  |
| miR160 | CL2867Contig1 | 1 | 469 | 488 | ARFR_ORYSJ | 188 | 1.00E-51 | Q653H7.1 |  |
| miR160 | CL3366Contig1 | 1 | 1128 | 1148 | SP1L1_ARATH | 114 | 8.00E-31 | B3H4F1.1 |  |
| miR164 | CL3713Contig2 | 2 | 160 | 179 | NAC22_ARATH | 209 | 8.00E-65 | Q84TE6.2 | ([Xie et al., 2000](#_ENREF_97); [Mallory et al., 2004](#_ENREF_58); [Song et al., 2010](#_ENREF_85); [Zeng et al., 2010](#_ENREF_102); [Li et al., 2011](#_ENREF_48)) |
| miR164 | Contig70 | 1.5 | 161 | 180 | GDL17_ARATH | 515 | 0 | Q8L5Z1.1 | - |
| miR166* | CL6185Contig1 | 1 | 484 | 504 | - | | | | - |
| miR166 | CL6185Contig1 | 0 | 1014 | 1033 | - | | | | - |
| miR166 | CL12710Contig1 | 1 | 1014 | 1033 | - | | | | - |
| miR166 | CL1528Contig1 | 1.5 | 138 | 157 | REV_ARATH | 1316 | 0 | Q9SE43.2 | ([Boualem et al., 2008](#_ENREF_10); [Carlsbecker et al., 2010](#_ENREF_12)) |
| miR166 | Contig17062 | 1 | 411 | 430 | ATHB8_ARATH | 1097 | 0 | Q39123.1 |  |
| miR166 | Contig433 | 1 | 540 | 560 | ATB14_ARATH | 747 | 0 | O04291.1 |  |
| miR166 | CL1528Contig1 | 1 | 138 | 157 | REV_ARATH | 1316 | 0 | Q9SE43.2 |  |
| miR166 | CL6185Contig1 | 2 | 996 | 1016 | - | | | | - |
| miR167 | Contig16373 | 1 | 191 | 210 | ARFQ_ORYSJ | 604 | 0 | Q653U3.1 | ([Kasschau et al., 2003](#_ENREF_38); [Allen et al., 2005](#_ENREF_2)) |
| miR167 | Contig1660 | 2 | 1799 | 1818 | ARAF_ARATH | 601 | 3.00E-99 | Q9ZTX8.2 |  |
| miR167 | F7JO6JY01BCFP7 | 1 | 192 | 211 | ARFF_ARATH | 189 | 5.00E-56 | Q9ZTX8.2 |  |
| miR167 | FS3H43Z02ESZ49 | 2 | 818 | 837 | ARFF_ARATH | 102 | 3.00E-25 | Q9ZTX8.2 |  |
| miR167 | FSG066401AQSV0 | 2 | 216 | 235 | ARFF_ARATH | 201 | 2.00E-60 | Q9ZTX8.2 |  |
| miR169 | Contig8712 | 2 | 352 | 371 | NFYA9_ARATH | 143 | 3.00E-38 | Q945M9.1 | ([Zhao et al., 2009](#_ENREF_105)) |
| miR169 | isotig19574 | 0 | 193 | 212 | NFYA9_ARATH | 143 | 2.00E-38 | Q945M9.1 |  |
| miR169 | Contig9977 | 0 | 191 | 211 | - | | | | - |
| miR169 | Contig8159 | 0.5 | 839 | 859 | NFYA3_ARATH | 138 | 5.00E-36 | Q93ZH2.2 | ([Zhao et al., 2009](#_ENREF_105)) |
| miR169 | F7JO6JY02IGHQK | 2 | 818 | 837 | - | | | | - |
| miR169 | Contig8712 | 0 | 193 | 212 | NFYA9_ARATH | 143 | 3.00E-38 | Q945M9.1 | ([Zhao et al., 2009](#_ENREF_105)) |
| miR169 | isotig19574 | 0 | 841 | 860 | NFYA9_ARATH | 143 | 2.00E-38 | Q945M9.1 |  |
| miR171 | CL313Contig1 | 2 | 819 | 838 | SCL6_ARATH | 100 | 1.00E-24 | O81316.1 | ([Llave et al., 2002](#_ENREF_53)) |
| miR171 | CL313Contig5 | 2 | 217 | 236 | SCL6_ARATH | 440 | 1.00E-143 | O81316.1 |  |
| miR171 | CL313Contig6 | 2 | 353 | 372 | SCL6_ARATH | 439 | 2.00E-143 | O81316.1 |  |
| miR171 | CL313Contig7 | 0 | 838 | 859 | SCL6_ARATH | 419 | 1.00E-139 | O81316.1 |  |
| miR171 | FS3H43Z01B1CT2 | 1 | 191 | 211 | - | | | | - |
| miR172 | CL1168Contig2 | 2 | 1380 | 1399 | AP2_ARATH | 297 | 8.00E-94 | P47927.1 | ([Aukerman and Sakai, 2003](#_ENREF_4); [Lauter et al., 2005](#_ENREF_46); [Jung et al., 2007](#_ENREF_37); [Mathieu et al., 2009](#_ENREF_60)) |
| miR172 | CL1887Contig1 | 2 | 342 | 361 | RAP27_ARATH | 137 | 3.00E-47 | Q9SK03.2 |  |
| miR172 | FSG066402DWMPE | 0 | 328 | 349 | - | | | | - |
| miR172 | CL2919Contig1 | 1.5 | 1184 | 1203 | RAP27_ARATH | 253 | 2.00E-78 | Q9SK03.2 | ([Aukerman and Sakai, 2003](#_ENREF_4); [Lauter et al., 2005](#_ENREF_46); [Jung et al., 2007](#_ENREF_37); [Mathieu et al., 2009](#_ENREF_60)) |
| miR172 | FS3H43Z02E08EH | 1.5 | 1134 | 1153 | - | | | | - |
| miR319 | CL9030Contig1 | 0.5 | 40 | 60 | - | | | | - |
| miR319 | FS3H43Z02DZ49P | 0.5 | 1170 | 1190 | - | | | | - |
| miR319 | CL1521Contig3 | 0.5 | 262 | 282 | - | | | | - |
| miR319 | Contig7196 | 0.5 | 360 | 380 | ZTP29_ARATH | 375 | 4.00E-127 | Q940Q3.1 | - |
| miR319 | CL9030Contig1 | 1 | 38 | 57 | - | | | | - |
| miR390 | Contig13750 | 0.5 | 262 | 281 | CAHC_PEA | 418 | 4.00E-143 | P17067.1 | - |
| miR390 | F7K499A01DBZED | 0.5 | 360 | 379 | MD37C_ARATH | 134 | 7.00E-37 | Q9LHA8.1 | - |
| miR390 | F7K499A02G9XVA | 0.5 | 1279 | 1298 | NLTP3_PRUDU | 75.1 | 1.00E-17 | Q43019.1 | - |
| miR390 | F7K499A02GC2IQ | 0.5 | 992 | 1012 | NLTP3_PRUDU | 75.1 | 1.00E-17 | Q43019.1 | - |
| miR394 | Contig5978 | 0.5 | 232 | 251 | FBX6_ARATH | 582 | 0 | Q9FZK1.1 | ([Song et al., 2012](#_ENREF_86)) |
| miR395 | FS3H43Z01CGA3M | 1.5 | 831 | 850 | - | | | | - |
| miR395 | FS3H43Z02ED1S5 | 1.5 | 80 | 99 | - | | | | - |
| miR395 | Contig16420 | 1 | 159 | 179 | APS1_ARATH | 744 | 0 | Q9LIK9.1 | ([Jones-Rhoades and Bartel, 2004](#_ENREF_35); [Allen et al., 2005](#_ENREF_2); [Kawashima et al., 2009](#_ENREF_39)) |
| miR396 | CL4830Contig2 | 0 | 159 | 179 | GRF4_ORYSJ | 81.6 | 6.00E-22 | Q6ZIK5.1 | ([Jones-Rhoades and Bartel, 2004](#_ENREF_35); [Liu et al., 2009](#_ENREF_52); [Rodriguez et al., 2010](#_ENREF_77)) |
| miR396 | CL6192Contig1 | 2 | 40 | 59 | GRF6_ORYSJ | 302 | 2.00E-93 | Q6AWY3.2 |  |
| miR397 | CL4847Contig2 | 1.5 | 52 | 71 | LAC17_ARATH | 499 | 1.00E-174 | Q9FJD5.1 | ([Jones-Rhoades and Bartel, 2004](#_ENREF_35)) ([Abdel-Ghany and Pilon, 2008](#_ENREF_1)) |
| miR397 | F7JO6JY02GWY6D | 2 | 13 | 32 | LAC10_ARATH | 70.9 | 3.00E-15 | Q6ID18.1 |  |
| miR397 | FS3H43Z02DKM7J | 0 | 159 | 178 | LAC12_ARATH | 101 | 5.00E-26 | Q9FLB5.1 |  |
| miR408 | Contig15929 | 2 | 1301 | 1320 | BCP_PEA | 80.1 | 1.00E-18 | Q41001.1 | ([Abdel-Ghany and Pilon, 2008](#_ENREF_1)) |
| miR408 | KIRST.18.C27 | 2 | 113 | 132 | EF1A_MANES | 92 | 3.00E-22 | O49169.1 | - |
| miR828 | CL13955Contig1 | 1 | 983 | 1002 | - | | | | - |
| miR828 | FS3H43Z02DGOM0 | 2 | 396 | 415 | - | | | | - |
| miR828 | CL5240Contig1 | 2 | 396 | 415 | MYBC_MAIZE | 169 | 2.00E-49 | P10290.1 | ([Hsieh et al., 2009](#_ENREF_32); [Lin et al., 2012](#_ENREF_51)) |
| miR828 | Contig17021 | 2 | 273 | 292 | WER_ARATH | 188 | 1.00E-57 | Q9SEI0.1 |  |
| miR828 | FSG066402D9IH2 | 2 | 396 | 415 | GL1_ARATH | 58.2 | 7.00E-11 | P27900.2 |  |
| miR828 | CL10173Contig1 | 1 | 644 | 663 | WER_ARATH | 188 | 1.00E-57 | Q9SEI0.1 |  |
