# Supplementary material for: Profiling microRNAs in Eucalyptus grandis reveals no mutual relationship between alterations in miR156 and miR172 expression and adventitious root induction during development
Source: BMC Genomics. 2014 Jun 25;15(1):524. doi: 10.1186/1471-2164-15-524 (PMC4094776; doi:10.1186/1471-2164-15-524)
Supplement: Supplementary file 3 — Additional file 3: Table S3: Predicted targets of E. grandis novel microRNAs discovered in this study. (DOCX 18 KB) [file 12864_2014_6229_MOESM3_ESM.docx]

| Target prediction | | | | | | Target BLAST | | | |
| --- | --- | --- | --- | --- | --- | --- | --- | --- | --- |
| miRNA  accession | Target accession | E value | | Target  start | Target  end | Target description | Score | E value | Swiss-Prot accession |
| Cluster_25933 | Contig8006 | 0 | 447 | | 468 | No significant similarity found | | | |
| Cluster_25933 | KIRST.33404.C1 | 1 | 148 | | 169 | No significant similarity found | | | |
| Cluster_25933 | CL2102Contig2 | 1 | 78 | | 99 | No significant similarity found | | | |
| Cluster_25933 | CL4761Contig1 | 1.5 | 57 | | 78 | NAC domain-containing protein 69 | 73.9 | 1e-15 | [Q9M126.1](http://www.ncbi.nlm.nih.gov/protein/75181714?report=genbank&log$=prottop&blast_rank=1&RID=0PX03BWF014) |
| Cluster_25933 | CL2817Contig2 | 2 | 153 | | 174 | [NAC transcription factor ONAC010](http://blast.ncbi.nlm.nih.gov/Blast.cgi#alnHdr_425936939) | 66.2 | 3e-13 | [A2YMR0.1](http://www.ncbi.nlm.nih.gov/protein/425936939?report=genbank&log$=prottop&blast_rank=1&RID=0PX03BWF014) |
| Cluster_31820 | Contig6460 | 0.5 | 529 | | 549 | No significant similarity found | | | |
| Cluster_31820 | Contig4316 | 2 | 1501 | | 1520 | Peroxisomal membrane protein 11D | 417 | 2e-142 | [O80845.2](http://www.ncbi.nlm.nih.gov/protein/75099949?report=genbank&log$=prottop&blast_rank=1&RID=0PX03BWF014) |
| Cluster_31821 | Contig6460 | 0.5 | 529 | | 549 | No significant similarity found | | | |
| Cluster_31821 | Contig4316 | 2 | 1501 | | 1520 | Peroxisomal membrane protein 11D | 417 | 2e-142 | [O80845.2](http://www.ncbi.nlm.nih.gov/protein/75099949?report=genbank&log$=prottop&blast_rank=1&RID=0PX03BWF014) |
| Cluster_31827_B | Contig6460 | 0.5 | 529 | | 549 | No significant similarity found | | | |
| Cluster_31827_B | Contig4316 | 2 | 1501 | | 1520 | Peroxisomal membrane protein 11D | 417 | 2e-142 | [O80845.2](http://www.ncbi.nlm.nih.gov/protein/75099949?report=genbank&log$=prottop&blast_rank=1&RID=0PX03BWF014) |
| Cluster_31829 | Contig6460 | 0.5 | 529 | | 549 | No significant similarity found | | | |
| Cluster_31829 | Contig4316 | 2 | 1501 | | 1520 | Peroxisomal membrane protein 11D | 417 | 2e-142 | [O80845.2](http://www.ncbi.nlm.nih.gov/protein/75099949?report=genbank&log$=prottop&blast_rank=1&RID=0PX03BWF014) |
| Cluster_31830_B | Contig6460 | 0.5 | 529 | | 549 | No significant similarity found | | | |
| Cluster_31830_B | Contig4316 | 2 | 1501 | | 1520 | Peroxisomal membrane protein 11D | 417 | 2e-142 | [O80845.2](http://www.ncbi.nlm.nih.gov/protein/75099949?report=genbank&log$=prottop&blast_rank=1&RID=0PX03BWF014) |
| Cluster_31845_B | Contig6460 | 0.5 | 529 | | 549 | No significant similarity found | | | |
| Cluster_31845_B | Contig4316 | 2 | 1501 | | 1520 | Peroxisomal membrane protein 11D | 417 | 2e-142 | [O80845.2](http://www.ncbi.nlm.nih.gov/protein/75099949?report=genbank&log$=prottop&blast_rank=1&RID=0PX03BWF014) |
| Cluster_31849_B | Contig6460 | 0.5 | 529 | | 549 | No significant similarity found | | | |
| Cluster_31849_B | Contig4316 | 2 | 1501 | | 1520 | Peroxisomal membrane protein 11D | 417 | 2e-142 | [O80845.2](http://www.ncbi.nlm.nih.gov/protein/75099949?report=genbank&log$=prottop&blast_rank=1&RID=0PX03BWF014) |
| Cluster_45808 | KIRST.33404.C1 | 1 | 148 | | 169 | No significant similarity found | | | |
| Cluster_45808 | CL2102Contig2 | 1 | 78 | | 99 | No significant similarity found | | | |
| Cluster_45808 | Contig8006 | 1 | 447 | | 468 | No significant similarity found | | | |
| Cluster_45808 | CL4761Contig1 | 1.5 | 57 | | 78 | NAC domain-containing protein 69 | 73.9 | 1e-15 | [Q9M126.1](http://www.ncbi.nlm.nih.gov/protein/75181714?report=genbank&log$=prottop&blast_rank=1&RID=0PX03BWF014) |
| Cluster_45808 | CL2817Contig2 | 2 | 153 | | 174 | [NAC transcription factor ONAC010](http://blast.ncbi.nlm.nih.gov/Blast.cgi#alnHdr_425936939) | 66.2 | 3e-13 | [A2YMR0.1](http://www.ncbi.nlm.nih.gov/protein/425936939?report=genbank&log$=prottop&blast_rank=1&RID=0PX03BWF014) |
| Cluster_45808 | CL2292Contig1 | 2 | 188 | | 207 | TMV resistance protein N | 140 | 2e-36 | [Q40392.1](http://www.ncbi.nlm.nih.gov/protein/46577339?report=genbank&log$=prottop&blast_rank=1&RID=0PX03BWF014) |
| Cluster_48252 | Contig8006 | 0 | 447 | | 468 | No significant similarity found | | | |
| Cluster_48252 | KIRST.33404.C1 | 1 | 148 | | 169 | No significant similarity found | | | |
| Cluster_48252 | CL2102Contig2 | 1 | 78 | | 99 | No significant similarity found | | | |
| Cluster_48252 | CL4761Contig1 | 1.5 | 57 | | 78 | NAC domain-containing protein 69 | 73.9 | 1e-15 | [Q9M126.1](http://www.ncbi.nlm.nih.gov/protein/75181714?report=genbank&log$=prottop&blast_rank=1&RID=0PX03BWF014) |
| Cluster_48252 | CL2817Contig2 | 2 | 153 | | 174 | [NAC transcription factor ONAC010](http://blast.ncbi.nlm.nih.gov/Blast.cgi#alnHdr_425936939) | 66.2 | 3e-13 | [A2YMR0.1](http://www.ncbi.nlm.nih.gov/protein/425936939?report=genbank&log$=prottop&blast_rank=1&RID=0PX03BWF014) |
