# Supplementary figures and images for: Profiling microRNAs in Eucalyptus grandis reveals no mutual relationship between alterations in miR156 and miR172 expression and adventitious root induction during development
Source: BMC Genomics. 2014 Jun 25;15(1):524. doi: 10.1186/1471-2164-15-524 (PMC4094776; doi:10.1186/1471-2164-15-524)

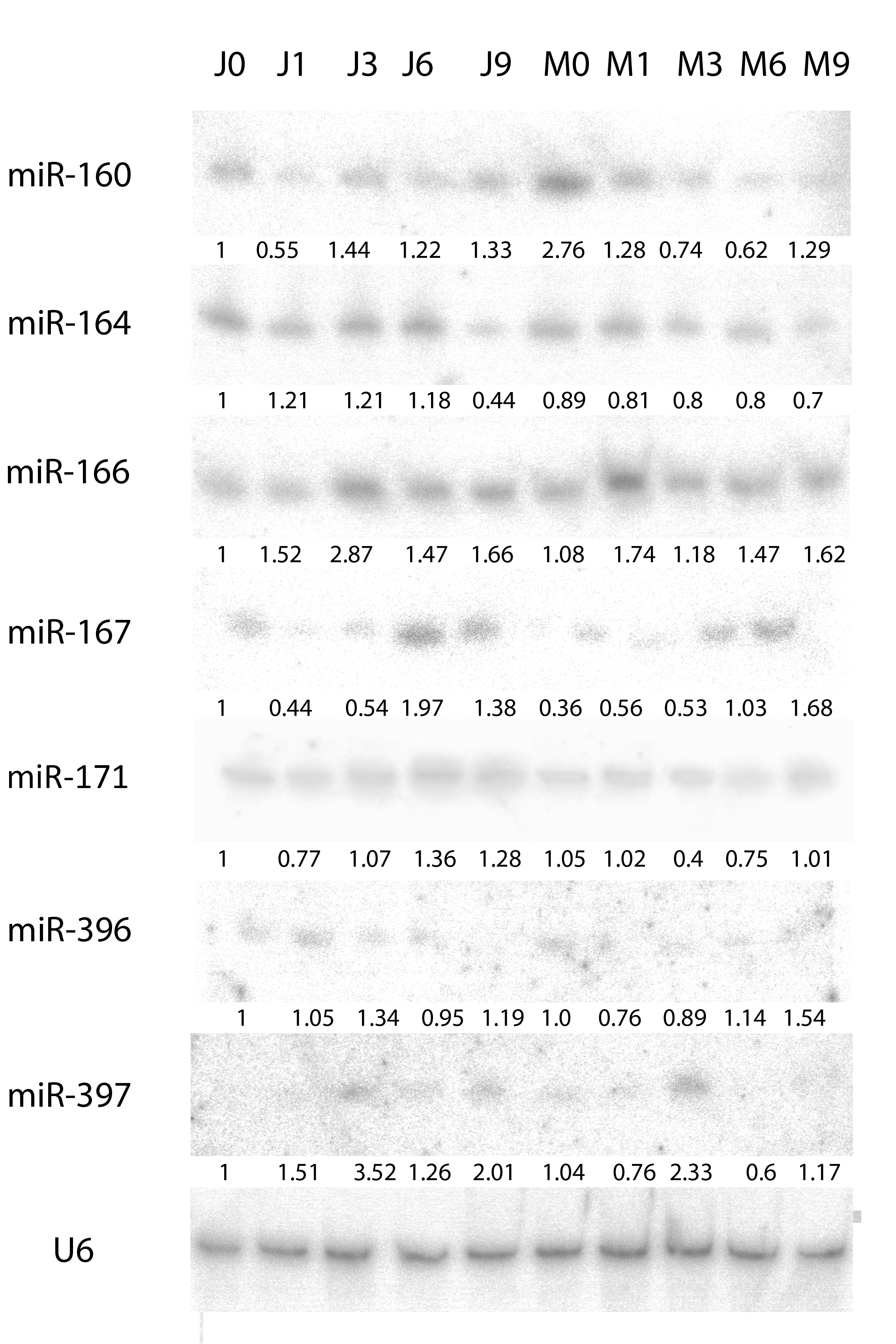

Supplement: Supplementary file 4 — Additional file 4: Figure S1: Levels of expression of microRNAs during adventitious root formation. (TIFF 7 MB) [file 12864_2014_6229_MOESM4_ESM.tiff]
