# Supplementary material for: Profiling microRNAs in Eucalyptus grandis reveals no mutual relationship between alterations in miR156 and miR172 expression and adventitious root induction during development
Source: BMC Genomics. 2014 Jun 25;15(1):524. doi: 10.1186/1471-2164-15-524 (PMC4094776; doi:10.1186/1471-2164-15-524)
Supplement: Supplementary file 5 — Additional file 5: Table S4: Levels of expression of predicted targets during adventitious root formation. (DOCX 21 KB) [file 12864_2014_6229_MOESM5_ESM.docx]

| **Putative target of miRNA** | **Sequence Accession** |  | **J0** | **J1** | **J3** | **J6** | **J9** | **M0** | **M1** | **M3** | **M6** | **M9** |
| --- | --- | --- | --- | --- | --- | --- | --- | --- | --- | --- | --- | --- |
|  | **[Top BLAST hit]** |  |  |  |  |  |  |  |  |  |  |  |
| 160 | CL2280Contig2  [*ARF10*] | AVG | 548 | 210 | 277 | 168 | 315 | 420 | 195 | 201 | 191 | 183 |
|  |  | SE | 47 | 30 | 35 | 43 | 59 | 64 | 40 | 16 | 36 | 27 |
|  |  | CLR | A | C | BC | C | BC | AB | C | C | C | C |
|  | CL2833Contig2 [*ARF17*] | AVG | 548 | 178 | 372 | 378 | 461 | 200 | 235 | 246 | 162 | 207 |
|  |  | SE | 131 | 28 | 88 | 28 | 64 | 25 | 61 | 20 | 45 | 30 |
|  |  | CLR | A | B | AB | AB | AB | B | B | AB | B | B |
|  | Contig2391  [*ARF18*] | AVG | 153 | 56 | 51 | 49 | 108 | 98 | 55 | 49 | 42 | 49 |
|  |  | SE | 30 | 7 | 7 | 11 | 25 | 20 | 5 | 2 | 10 | 5 |
|  |  | CLR | A | B | B | B | AB | AB | B | B | B | B |
| 164 | *NAC1* | AVG | 18 | 37 | 127 | 202 | 228 | 69 | 52 | 81 | 70 | 121 |
|  |  | SE | 1.1 | 6 | 16 | 43 | 28 | 17 | 2 | 9 | 25 | 27 |
|  |  | CLR | C | BC | AB | A | A | BC | BC | BC | BC | AB |
| 166 | CL1528Contig1  [*HD-ZIP protein REV*] | AVG | 1674 | 796 | 598 | 181 | 726 | 1421 | 621 | 510 | 609 | 525 |
|  |  | SE | 341 | 147 | 88 | 22 | 210 | 311 | 78 | 48 | 136 | 38 |
|  |  | CLR | A | ABC | BC | C | BC | AB | BC | BC | BC | BC |
| 167 | Contig1660  [*ARF6*] | AVG | 1624 | 596 | 1043 | 806 | 1016 | 843 | 1061 | 935 | 481 | 484 |
|  |  | SE | 322 | 59 | 235 | 36 | 203 | 153 | 285 | 95 | 83 | 122 |
|  |  | CLR | A | B | AB | AB | AB | AB | AB | AB | B | B |
| 171 | CL313Contig7  [*SCL6*] | AVG | 334 | 155 | 190 | 60 | 234 | 251 | 110 | 162 | 172 | 135 |
|  |  | SE | 59 | 37 | 102 | 39 | 94 | 16 | 30 | 5 | 15 | 24 |
|  |  | CLR | A | AB | AB | B | AB | AB | B | AB | AB | AB |
| 397 | CL4847Contig2  [*LAC17*] | AVG | 81 | 37 | 20 | 9 | 75 | 139 | 6 | 41 | 23 | 53 |
|  |  | SE | 30 | 6 | 1 | 3 | 9 | 33 | 3 | 9 | 6 | 43 |
|  |  | CLR | AB | B | B | B | AB | A | B | B | B | AB |

Expression analysis of miR predicted target. The table shows Nanostring reading counts of miRNA of putative targets from total RNA samples extracted from juvenile (J) and mature (M) *E. grandis* cuttings. Cuttings were sampled before AR induction (0) and 1, 3, 6, and 9 days after induction. AVG – reads average, SE - ±1 Standard Error, CLR – Connecting Letter Report, levels not connected by the same letter are significantly different (all pairs Tukey HSD, *P* < 0.05)
